# Heat Acclimation Decay and Re-Induction: A Systematic Review and Meta-Analysis

**DOI:** 10.1007/s40279-017-0808-x

**Published:** 2017-11-11

**Authors:** Hein A. M. Daanen, Sebastien Racinais, Julien D. Périard

**Affiliations:** 1 Department of Human Movement Sciences, Faculty of Behavioural and Movement Sciences, Amsterdam Movement Sciences, Vrije Universiteit Amsterdam, Van der Boechorststraat 7, 1081BT Amsterdam, The Netherlands; 2 Athlete Health and Performance Research Centre, Aspetar Orthopaedic and Sports Medicine Hospital, Doha, Qatar; 3 Research Institute for Sport and Exercise, University of Canberra, Canberra, ACT Australia

## Abstract

**Background:**

Although the acquisition of heat acclimation (HA) is well-documented, less is known about HA decay (HAD) and heat re-acclimation (HRA). The available literature suggests 1 day of HA is lost following 2 days of HAD. Understanding this relationship has the potential to impact upon the manner in which athletes prepare for major competitions, as a HA regimen may be disruptive during final preparations (i.e., taper).

**Objective:**

The aim of this systematic review and meta-analysis was to determine the rate of HAD and HRA in three of the main physiological adaptations occurring during HA: heart rate (HR), core temperature (*T*_c_), and sweat rate (SR).

**Data Sources:**

Data for this systematic review were retrieved from Scopus and critical review of the cited references.

**Study Selection:**

Studies were included when they met the following criteria: HA, HAD, and HRA (when available) were quantified in terms of exposure and duration. HA had to be for at least 5 days and HAD for at least 7 days for longitudinal studies. HR, *T*_c_, or SR had to be monitored in human participants.

**Study Appraisal:**

The level of bias in each study was assessed using the McMaster critical review form. Multiple linear regression techniques were used to determine the dependency of HAD in HR, *T*_c_, and SR from the number of HA and HAD days, daily HA exposure duration, and intensity.

**Results:**

Twelve studies met the criteria and were systematically reviewed. HAD was quantified as a percentage change relative to HA (0% = HA, 100% = unacclimated state). Adaptations in end-exercise HR decreased by 2.3% (*P* < 0.001) for every day of HAD. For end-exercise *T*_c_, the daily decrease was 2.6% (*P* < 0.01). The adaptations in *T*_c_ during the HA period were more sustainable when the daily heat exposure duration was increased and heat exposure intensity decreased. The decay in SR was not related to the number of decay days. However, protracted HA-regimens seem to induce longer-lasting adaptations in SR. High heat exposure intensities during HA seem to evoke more sustained adaptations in SR than lower heat stress. Only eight studies investigated HRA. HRA was 8–12 times faster than HAD at inducing adaptations in HR and *T*_c_, but no differences could be established for SR.

**Limitations:**

The available studies lacked standardization in the protocols for HA and HAD.

**Conclusions:**

HAD and HRA differ considerably between physiological systems. Five or more HA days are sufficient to cause adaptations in HR and *T*_c_; however, extending the daily heat exposure duration enhances *T*_c_ adaptations. For every decay day, ~ 2.5% of the adaptations in HR and *T*_c_ are lost. For SR, longer HA periods are related to better adaptations. High heat exposure intensities seem beneficial for adaptations in SR, but not in *T*_c_. HRA induces adaptations in HR and *T*_c_ at a faster rate than HA. HRA may thus provide a practical and less disruptive means of maintaining and optimizing HA prior to competition.

## Key Points

**Table:** 

Five or more heat acclimation (HA) days induce stable adaptations in end-exercise heart rate and core temperature (*T*_c_), but for every day without heat exposure (i.e., decay day) ~ 2.5% of these adaptations are lost.
Extending daily heat exposure duration improves adaptations in *T*_c_.
For sweat rate, longer HA periods are related to more sustainable adaptations.

## Introduction

When acutely exercising in hot environmental conditions, whole-body temperature increases, as does the cardiovascular response and perceptions of exertion and thermal discomfort, leading to impairments in endurance performance (e.g., Périard et al. [1], Périard and Racinais [2]). However, chronic exposure to heat stress enhances thermoregulatory responses [3, 4], improves submaximal exercise performance [5], increases maximal aerobic capacity (maximum oxygen uptake [VO_2max_]) [6, 7], and improves thermal comfort [8–10] in the heat. The benefits of heat acclimation (HA) are achieved through enhanced sweating and skin blood flow responses, plasma volume expansion, better fluid balance (i.e., hydration) and cardiovascular stability, and acquired thermal tolerance [11–15]. The time course of HA is remarkably rapid, with a significant fraction of the benefits associated with HA occurring during the first week of heat exposure [16, 17], and the remaining thermoregulatory benefits generally being complete or optimized after 10–14 days [12, 14, 15, 18]. The process of adaptation related to HA can occur in artificial/laboratory settings (i.e., heat acclimation) and from exposure to natural environments (i.e., heat acclimatization) [19, 20]. For the purposes of the review, the abbreviation HA will be used to represent both types of induction.

Athletes expecting to compete in hot conditions, such as the Tokyo 2020 Summer Olympics, should include HA in their training program to optimize performance [21, 22]. If HA is not induced, performance in the heat can be expected to be suboptimal [23–25]. Therefore, it is recommended to acclimate for 2 weeks preceding a major event in the heat [14]. However, athletes also need to taper prior to competition [26]. The stepwise recovery during tapering is essential to enable the consolidation of physiological adaptations after a period of strenuous training, and HA should not interfere with this process. Indeed, HA should complement or be conducted prior to the taper period. Therefore, it would be of benefit if HA decay (HAD) was limited and/or if heat re-acclimation (HRA) sessions could be performed without interfering with the taper. HRA sessions could be conducted as active or passive interventions, depending on the state of HA and individual circumstances, thereby minimizing disruptions to training.

This systematic review and meta-analysis evaluates the time course of HAD and HRA and places them in the context of elite sport. To appreciate these processes, a brief examination of HA induction pathways, guidelines, and quantification is first performed.

### Heat Acclimation Induction Pathways

**Fig. 1:**
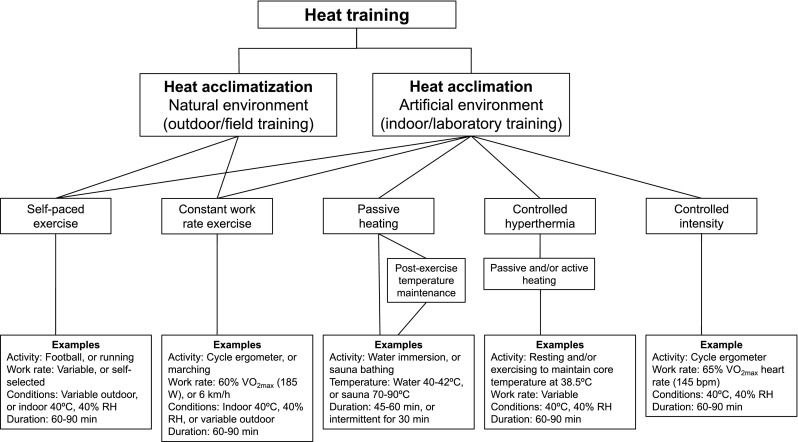
Schematic overview of methods for heat acclimation and heat acclimatization, with examples. Various combinations of temperature and humidity are possible, as well as the use of portable heaters and wearing additional clothing. *RH* relative humidity, *VO*_*2max*_ maximal oxygen uptake

The HA phenotype is generally achieved through four broad induction pathways: (i) constant work rate exercise [27–30]; (ii) self-paced exercise [31, 32]; (iii) controlled hyperthermia, or isothermic HA [33–36]; and (iv) the recently proposed controlled heart rate (HR) (i.e., relative intensity) approach [12, 15] (Fig. 1).

The repeated exercise–heat exposure at a constant work rate approach has traditionally been utilized in occupational and military settings [37–41] and has been demonstrated to effectively induce adaptation and improve performance in the heat [7, 28, 29, 42]. However, it has been argued that the maintenance of a constant forcing function by fixing endogenous and exogenous thermal loading leads to a decrease in physiological strain as adaptations progressively develop, thus attenuating the potential for further adaptation [13, 43]. Similarly, the self-paced HA approach was initially implemented by the military to evaluate its effectiveness and safety during recruit training and for preparing unacclimatized soldiers for rapid deployment to hot environments [31, 32]. More recently, self-paced HA was utilized during an outdoor training camp in trained cyclists [5, 44] and it is typically the approach adopted when conducting HA with team-sport athletes [45–49].

In contrast, controlled hyperthermia or isothermic HA regimens, originally developed by Fox et al. [33, 50, 51], are purported to provide a forcing function that increases in proportion to adaptations by manipulating endogenous and/or exogenous thermal loads to reach and sustain a target core temperature (*T*_c_) (e.g., 38.5 °C) [13, 52]. Relative to the constant work and self-paced HA approaches, greater physiological adaptations should occur during a given period with controlled hyperthermia. However, controlled hyperthermia HA usually involves a balance of passive (i.e., resting) and active (i.e., exercise) heating to attain and maintain the target *T*_c_. Although passive heat exposure results in some adaptation [53–56], based on the principle of training specificity [13], exercise-induced HA is likely to be more effective in developing sport-specific adaptations [12]. Interestingly, similar magnitudes of enhancement in exercise performance and capacity were recently reported when comparing the controlled hyperthermia and constant work rate HA literature [14]. The comparable response between regimens may stem in part from different HA approaches (i.e., stimuli) inducing distinctive autonomic responses that result in comparable physiological adaptations. For example, Moran et al. [57] demonstrated in rats that exercise training and HA improved cardiac efficiency during exercise under heat stress via different pathways. Whilst exercise training had a more pronounced effect on HR, HA attenuated the increase in blood pressure [57]. The similarity in responses in humans may also stem in part from the controlled hyperthermia stimulus inducing thermoregulatory adaptations, but perhaps failing to fully produce exercise-specific modifications [58] due to the passive or intermittent (i.e., passive and active) nature of the regimen. Instances of this are reflected by the maintenance of lower absolute and relative exercise work rates during controlled hyperthermia sessions than with constant work rate HA [59, 60], as well as relatively low cardiovascular responses during daily exposure (e.g., mean HR of ~ 122 beats/min) [61, 62] and decreasing mean HR throughout acclimation [59, 63].

An alternative approach to HA lies with passive heat exposure following exercise in temperate conditions. This can be achieved via sauna exposure [64, 65] or hot water immersion [66] to maintain and induce a slight increase in whole-body temperature after a training session in cool conditions. Whilst these studies have demonstrated positive adaptations related to changes in blood volume, it remains to be determined whether passive heat exposure after exercise yields adaptations similar to those of exercise-HA. Notwithstanding, this approach may provide a more practical means of implementing HA in high-level athletes facing difficulties with adopting traditional exercise-based HA regimens [67].

As recently highlighted in a HA meta-analysis, the maintenance of a greater relative exercise intensity may hasten HA adaptations [14]. Périard et al. [12, 15] have proposed a controlled exercise intensity HA approach, whereby a given level of cardiovascular strain is maintained during acclimation to optimize adaptations. The level of strain achieved and sustained would correspond to the HR associated with a predetermined relative exercise intensity. This HR would then be sustained during the HA regimen with adjustments in work rate occurring as adaptations develop, thus providing a constant cardiovascular stimulus. A recent study showed that a short (5 days) controlled HR HA regimen induced similar adaptations to training in temperate conditions while having a 30% lower mechanical load [68].

### Guidelines for Duration, Intensity, and Environmental Conditions

Although it is well-accepted that moderate to long-term HA (> 8 days) is more beneficial than short-term HA (< 8 days) at inducing adaptation [12, 14, 15, 18, 52, 69], no clear recommendations currently exist on the precise duration for each heat exposure session [21, 22]. This relates to the magnitude of physiological adaptations induced by HA depending on several factors, including the intensity, duration, frequency, and number of heat exposures [12, 70].

Lind and Bass [39] demonstrated over 50 years ago that exercise-HA (i.e., walking in ~ 48.9 °C conditions) for 100 min/day was more beneficial at inducing adaptation than one or two 50 min daily exposures, and that two 100 min daily exposures resulted in no additional benefit. This absence of any advantage for twice-daily protocols was recently confirmed by Willmott et al. [71]. Based on the work of Lind and Bass [39], Pandolf [16] proposed that 100 min of daily exercise in the heat seemed optimal for eliciting heat adaptation, and several studies have thus utilized this timeframe [7, 31, 37, 72–75]. In a recent consensus statement on training and competing in the heat [22], it was suggested that athletes planning to compete in hot ambient conditions should exercise–heat acclimate for at least 60 min/day. This recommendation was contingent on HA inducing a meaningful increase in whole-body temperature (i.e., core and skin), and strongly stimulating the sweating response. As such, this timeframe should be taken in context and extended, if required, to ensure sufficient thermal strain and stimulus for adaptation. Moreover, given the diversity in the various HA approaches currently being utilized, regimen-specific data concerning the optimal daily exposure time remain to be determined. Nevertheless, an intermittent HA protocol (ten sessions in 3 weeks) has been shown to lead to suboptimal adaptation relative to a regimen involving daily heat exposure [23]. Moreover, performing exercise in the heat once every week does not lead to any adaptations in HR, rectal temperature (*T*_re_), sweat rate (SR), and oxygen uptake [76].

The prescription of exercise intensity during HA is a parameter influenced by several factors, including time, both for daily exposure and regimen duration, as well as training cycle (e.g., taper period). A study by Houmard et al. [77] demonstrated that low-intensity long-duration (60 min at 50% VO_2max_) exercise elicited analogous HA benefits to that of moderate-intensity short-duration (30–35 min at 75% VO_2max_) exercise. Whilst prescribing a greater relative exercise intensity may facilitate HA when constrained by time, absolute work load should be increased over the HA period to ensure the adaptation stimulus remains constant. Importantly, the stimulus (i.e., endogenous and/or exogenous thermal load) should elicit an increase in whole-body temperature and sweating for a sustained period to induce optimal adaptation within a given timeframe [21, 22].

When undertaking HA in laboratory or artificial settings, environmental temperature is typically set to 40 °C [14]. Based on the conditions in which competition is to take place, relative humidity may be manipulated to produce hot/dry or hot/wet conditions. HA in hot/dry conditions has been shown to improve exercise in hot/wet conditions, relative to no HA [33, 78], and vice versa [79]. Interestingly, Griefahn [80] observed no difference in HA adaptation between warm/humid, hot/dry, and radiant heat climates when similar wet bulb globe temperatures (WBGTs) were elicited. However, the physiological and biophysical differences between dry and humid heat suggest that somewhat different physiological adaptations may develop [12, 81]. For example, Patterson et al. [82] reported interregional variations in the capacity to increase local SR following humid HA, whereas Sawka et al. [83] demonstrated that HA in hot/humid conditions elicits lower SRs than in a hot/dry environment. Accordingly, simulating conditions similar or slightly more stressful than those that are to be encountered during competition may be recommended. Ultimately, HA in the outdoor environment in which an event will occur is likely to induce adaptations specific to that setting [12].

### Quantifying Heat Acclimation

HA induces central and peripheral adaptations that elicit changes in plasma volume, HR, exercise capacity, thermal comfort, mean skin temperature (*T*_sk_) and *T*_c_, and SR. The quantification of these individual adaptations in both magnitude and timeframe is highly variable and strongly associated with the HA approach utilized. It also may be influenced by fitness [84–86], age [16, 87, 88], and sex [89, 90]. Although formulae have been developed and utilized to calculate the percentage loss of HA during the decay period [75, 91, 92], adaptations related to the induction of HA are typically evaluated in relation to the attainment of a plateau in particular responses [75, 80, 91, 93–95]. Therefore, as adaptations develop during a particular HA regimen, the presence of a sustained plateau in various parameters indicates the success, or failure, of that intervention. It should be noted that the attainment of a plateau during a particular HA regimen does not imply that additional improvements in certain parameters cannot be achieved. Indeed, environmental conditions and/or exercise intensity may be manipulated to potentially provide a greater stimulus for adaptation.

### Heat Acclimation Decay and Re-Induction

Although much work has been done regarding the induction of HA, few data are available on HAD and HRA. Armstrong and Maresh [96] dedicated two paragraphs to decay in their review on HA and pointed to the idiosyncrasy of the time course of HAD. Pandolf [16] reviewed eight studies from 1943 to 1963 and observed that these studies were rather limited due to small sample sizes [30, 78, 79, 97], incomplete HA [39, 98], or inappropriate measurements [99, 100]. The general picture was that HA was better retained after hot/dry acclimation than after wet/humid acclimation and by men that remained physically fit. HA persisted for at least 1 week, but a major portion was lost after 1 month. Pandolf [16] noted that the published literature was far more robust and plentiful on the time course of HA than its decay. A more recent review focusing on short-term HA and its decay concluded that decay in adaptations related to HR occurred faster than those relating to *T*_c_ [52]. However, the authors indicated that limited research was available over the decay timeframe.

Given the limited knowledge regarding the implementation timeframe to optimize athletic performance via HA, along with the availability of additional studies examining HAD and HRA [42, 75, 101–103], an updated review regarding these aspects is warranted. The primary aim of the current systematic review was to determine the time course of HAD and HRA on cardiovascular function (i.e., HR), *T*_c_ changes, and SR. A secondary aim was to provide practical guidelines for the optimization of preparation for performing in the heat in consideration of the HA adaptive period.

## Methods

### Data Sources

Scopus, the largest abstract and citation database of peer-reviewed literature, was searched for available literature from its inception through to 2 August 2017. The search strategy included the following terms for the title, abstract, and keywords: ‘heat’, ‘acclim*’, ‘decay’, ‘temperature’, and ‘human’. Replacing ‘acclim*’ and ‘decay’ with ‘reacclim*’ did not yield additional articles. Searches were limited to the English language only. The references of all relevant articles were examined for further articles.

### Study Selection

One reviewer scanned the titles for duplicates and read the abstracts to determine the relevance for this review. If relevant, the full-text was retrieved and shared between the authors of this review. The eligibility criteria were English abstract, human participants, completed HA with decay period quantified in terms of exposure and duration, and data on HR, SR, or body temperatures available in the paper. No restriction was used for the publication format: articles, reports, or conference proceedings were all accepted as a source.

### Outcome Measures

The data on HR, *T*_c_, skin temperature, SR, performance, and thermal sensation (TS) from the selected studies before HA, after HA, and during HAD and HRA served as input for further analysis. HAD was quantified using the formula developed by Williams et al. [92] and modified by Pandolf et al. [91] (Eq. 1):

1 $$ \% {\text{ Decay}} = 100 \times \frac{{{\text{Acclimatized value }} - {\text{Value after decay}}}}{{{\text{Acclimatized value}} - {\text{Unacclimatized value}}}}. $$

**Fig. 2:**
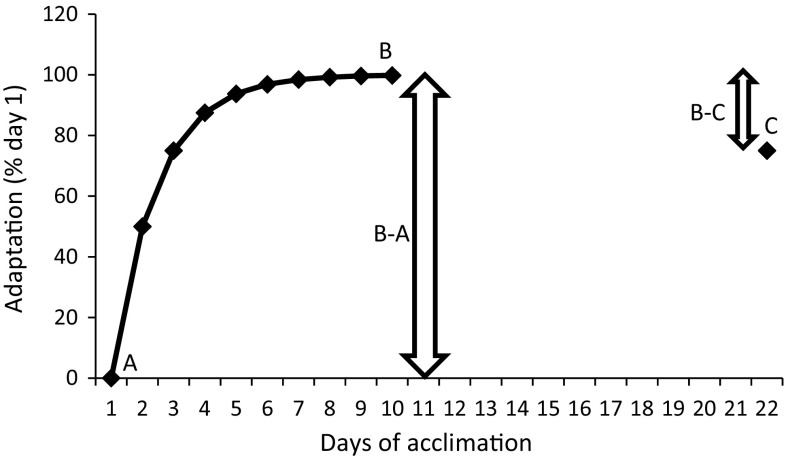
Example of the heat acclimation decay calculation. A is the heat unacclimated state, B is the acclimated state, and C is the status on return following a decay period without heat exposure (e.g., 12 days after the final day of heat acclimation).  % Decay is defined as (B–C)/(B–A)

Figure 2 provides an example of HAD for an arbitrary variable (e.g., HR). Following a HA period of 10 days, participants return on day 22. HAD (B–C) represents ~ 25% in this example. If C equaled 100, then complete adaptation would remain and HAD would equate to 0%. If C returned to level A, the adaptation would be completely lost and HAD would equate to 100%.

**Fig. 3:**
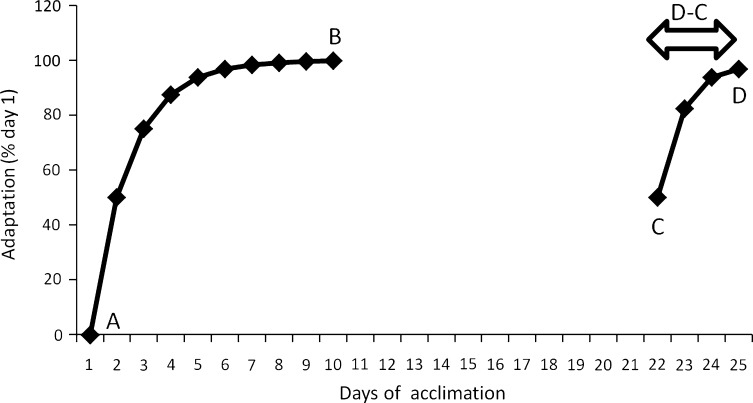
Example of re-acclimation quantification. A is the unacclimated state, B is the acclimated state, C is the status on return following a decay period without heat exposure, and D is the attainment of a status similar to that following heat acclimation (i.e., B)

There is no consensus on how to determine HRA status. However, most studies consider the number of days to reach prior full acclimation status as the best parameter (Fig. 3). In other words, when D equals B, the difference between D and C is the indicator for complete HRA: the number of days to get back to the status of full HA.

To quantify the relationship between three of the main adaptations occurring during HA and their rate of decay, stepwise multiple regressions were performed using Statistica™ (Dell Inc., Round Rock, TX, USA) [104] with HR decay (%), *T*_c_ decay (%), and SR decay (%) as dependent variables and number of HA days and HAD days, daily exposure duration, and daily heat exposure intensity, assessed as WBGT-value, as independent variables.

### Risk of Bias Assessment and Data Extraction

The full-text articles were read by the authors and the level of bias was assessed by each author independently using the McMaster clinical review form^1^ and later summarized in a consensus meeting.

**Fig. 4:**
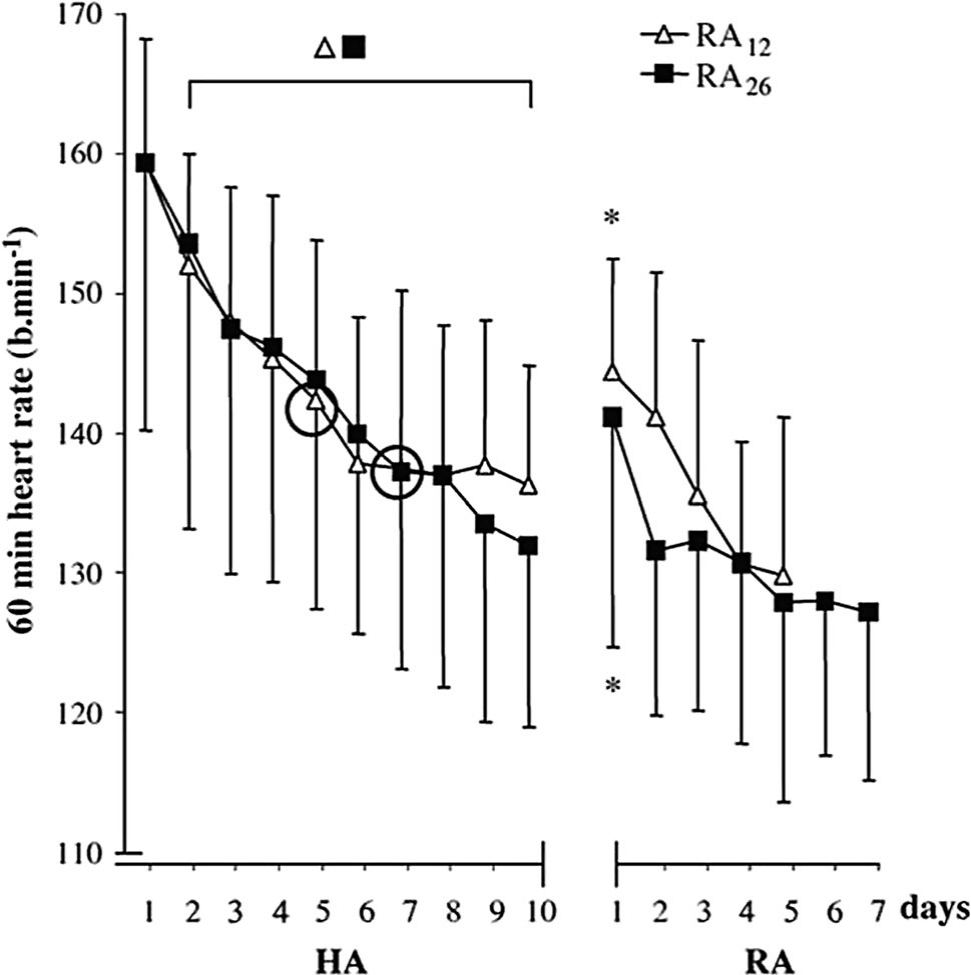
Example of a cross-sectional research protocol for evaluating heat acclimation (HA) decay. Heart rate (mean ± standard deviation) for eight participants who re-acclimated (RA) for 5 days starting 12 days after a 10-day HA period (RA_12_) and eight participants that re-acclimated for 7 days starting 26 days after a 10-day HA period (RA_26_). Circles indicate the day after which there were no significant changes. Asterisks denote significant differences from the mean of the end of the HA period (day 8–10). Reproduced with permission from Weller et al. [75]

Two study designs are typically used to investigate HAD: transversal and longitudinal studies. In the transversal (cross-sectional) design, two or more heat-acclimated groups are tested at different decay time intervals. An example of such a protocol is shown in Fig. 4. In this study, one group of participants returned 12 days after HA and the other group after 26 days [75]. Using this approach, the risk of partial HRA is avoided, but it is important that both groups are comparable in terms of HA status prior to and immediately after the HA regimen. Consequently, it is important that parameters that can influence HAD, such as physical fitness or regular training, are taken into account when composing the participant groups. The longitudinal design involves a single HA group in which HAD is tested multiple times. The advantage of this study design is that the changes in physiological responses to heat can be evaluated within participants. However, the obvious limitation is that a heat stress test may interrupt HAD and can be seen as an HRA session. Notwithstanding, both types of studies have been included in the interpretation.

## Results

### Literature Search Results

#### Study Description

**Fig. 5:**
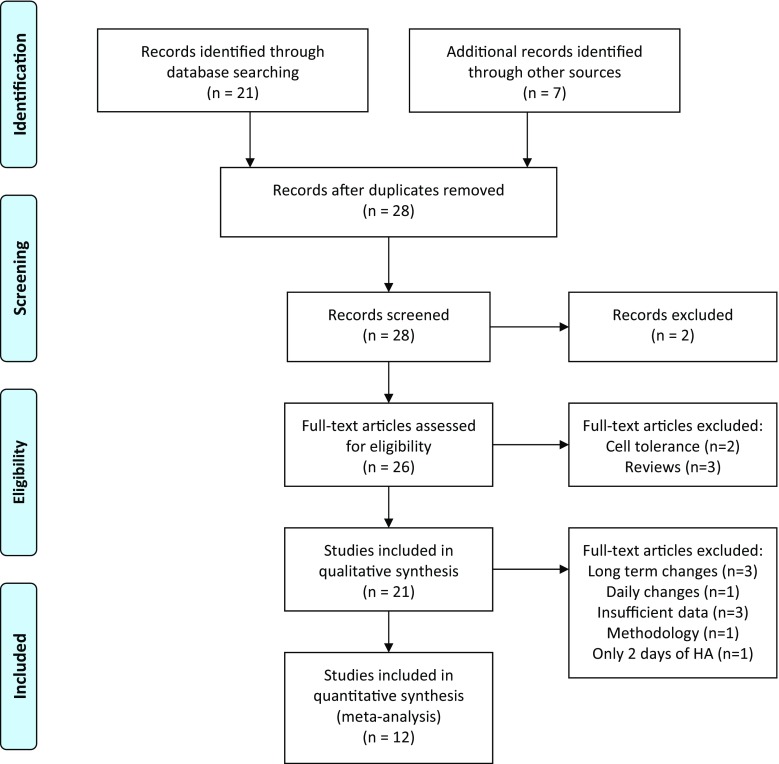
PRISMA (Preferred Reporting Items for Systematic Reviews and Meta-Analyses) flow diagram outlining the identification and inclusion processes for the qualitative and quantitative reviews [119]. *HA* heat acclimation

The literature search resulted in the identification of 21 papers (Fig. 5). A critical review of the references and additional reading led to the inclusion of seven additional studies: six early studies [39, 78, 92, 98–100] and a recent paper focusing on the effect of dehydration on HA and HAD [105]. Two papers were excluded since they were not relevant to this review (i.e., one was on an eye disease and one on cold baths)—those are the two excluded records in Fig. 5. Of the remaining 26 papers, five full-text articles were excluded: two dealt with cell tolerance to heat [11, 106] and three were reviews on HAD [16, 96] or HA [13]. The paper by Horowitz [11] provides an excellent overview of the cellular adaptations related to HA, and as such cellular adaptations are not discussed in the review. Thus, a total of 21 full-text papers were investigated. Table 1 shows the number of participants and their characteristics extracted from the 21 studies included in this review of HAD and HRA. Females were noticeably underrepresented and all participants were of young age. The studies of Poirier et al. [42] and Flouris et al. [103] describe different variables from the same experiment. Flouris et al. [103] focus on HR variability, while Poirier et al. [42] describe HR, temperature, and fluid loss in more detail. Both studies were therefore included in the analysis.

**Table 1:** Studies evaluating heat acclimation decay and re-acclimation included in the review

Study	Males	Females	Type of study	Age [years (mean or range)]	VO_2max_ (mL/min/kg)
Adam et al. [99]	16		Longitudinal	20	
Ashley et al. [102]	5	5	Cross-sectional and longitudinal	31	34
Bean and Eichna [78]	7		Cross-sectional	17–43	
Daanen et al. [93]	15		Longitudinal	24	55
Flouris et al. [103]	10		Longitudinal	23	51
Garrett et al. [34]	10		Longitudinal	28	57
Gill and Sleivert [23]	4	10	Short interval	24	48
Henschel et al. [98]	24		Cross-sectional	19–28	
Lee et al. [107]	28/35^a^		Population	29/26^b^	
Lee et al. [108]	13/11^a^		Population	31/22^b^	
Lind and Bass [39]	16		Cross-sectional		
Neal et al. [105]	8		Cross-sectional	21	57
Pandolf et al. [91]	24		Cross-sectional	22	50
Pichan et al. [111]	12		Cross-sectional	23	Fit
Poirier et al. [42]	10		Longitudinal	23	51
Saat et al. [101]	16		Longitudinal	18	51
Stephens and Hoag [110]		6	Longitudinal	18–30	39
Weller et al. [75]	16		Cross-sectional	28	52
Wijayanto et al. [109]	12/12^a^		Population	25	
Williams et al. [92]	51		Cross-sectional		
Wyndham et al. [41]	73		Cross-sectional		
Total	428	21			

*VO*_*2max*_ maximum oxygen uptake

^a^Number of tropical indigenous participants/number of temperate indigenous participants

^b^Mean age of tropical indigenous participants/mean age of temperate indigenous participants

Of the 21 studies retained for the review, 12 studies were suitable for a quantitative analysis of decay. The following studies were not included:

- Three studies investigated HAD for a population of heat-acclimated indigenous individuals who moved to a relatively cold region [107–109].
- One study investigated the effects of daily versus once every other day HA [23]. This study is included in the qualitative review since one can argue that 1 day of HAD is compared with no decay.
- Three studies contained insufficient data for a quantitative analysis [78, 99, 100].
- One study was excluded since a different population was used for the determination of the acclimated and unacclimated state [92].
- Finally, one study was excluded as the HA period comprised only 2 days [98].

Stepwise multiple linear regression analyses were performed using 12 studies, two of which described different variables of the same experiment [42, 103]. Both transversal and longitudinal studies were included, leading to a total of 29 datapoints for HR, 27 for *T*_c_, and 13 for SR. SR was not measured in two studies [34, 102] and was considered too variable for interpretation in another study [39]. Certain studies were excluded from the analysis as they observed no changes in *T*_c_ [102] or SR [91, 110] during HA, and as such decay could not be calculated. The longitudinal studies were included when the heat stress tests were at least a week apart to allow for sufficient recovery. For the longitudinal studies, the decay periods were 14 [102], 11 [93, 101], 7 [34, 42], and 12 and 16 days [110]. For the studies included in the quantitative analysis, the number of HA days varied from 5 to 14, the WBGT (a combined measure of temperature and humidity) ranged from 26 to 36 °C and the duration from 60 to 120 min. The number of participants ranged from 6 [110] to 15 [93] (Table 1). The exclusion criteria were kept to a minimum in an attempt to include as many studies as possible, despite some studies having shortcomings (discussed in Sect. 3.1.2).

#### Risk of Bias and Limitations

The results of the assessment using the McMasters critical appraisal tool are shown in Table 2. All included studies, except the abstract of Adam et al. [99], gave a rationale for the study and provided clear conclusions. Control groups were used in different ways. For example, some compared heat-exposed participants to a control group of cold-exposed participants [99], while others compared the effects of exercise in the decay period to a control group without exercise [101]. The other controlled studies employed different decay periods for comparison. Four studies included too few participants to perform reliable statistics, but the observations may still be of interest [39, 78, 102, 110].

**Table 2:** Overview of the McMaster risk of bias assessment of the selected studies

Study	Study purpose	Study design		Sample	Reliability and validity	Intervention	Statistical analysis	Conclusions	Total (/8)
		Controlled	Randomized						
Adam et al. [99]	0	1	1	1	0	1	1	1	6
Ashley et al. [102]	1	1	1	0	1	1	1	1	7
Bean and Eichna [78]	1	0	0	0	0	1	0	1	3
Daanen et al. [93]	1	0	0	1	1	1	1	1	6
Flouris et al. [103]	1	0	0	1	1	1	1	1	6
Garrett et al. [34]	1	0	0	1	1	1	1	1	6
Gill and Sleivert [23]	1	1	1	1	1	1	1	1	8
Henschel et al. [98]	1	0	0	1	0	1	0	1	4
Lee et al. [107]	1	1	0	1	1	1	1	1	7
Lee et al. [108]	1	1	0	1	1	1	1	1	7
Lind and Bass [39]	1	0	0	0	0	1	0	1	3
Neal et al. [105]	1	1	1	1	1	1	1	1	8
Pandolf et al. [91]	1	1	1	1	1	1	1	1	8
Pichan et al. [111]	1	0	0	1	1	1	1	1	6
Poirier et al. [42]	1	0	0	1	1	1	1	1	6
Saat et al. [101]	1	1	1	1	1	1	1	1	8
Stephens and Hoag [110]	1	0	0	0	0	1	1	1	4
Weller et al. [75]	1	1	1	1	1	1	1	1	8
Wijayanto et al. [109]	1	1	0	1	1	1	1	1	7
Williams et al. [92]	1	1	1	1	1	1	1	1	8
Wyndham et al. [41]	1	0	0	1	1	1	1	1	6

### Heat Acclimation Decay Studies

Table 3 provides an overview of the type of study, the ways via HA was achieved, and the characteristics of the heat stress tests, while Table 4 gives an overview of the measured variables. Most studies included three physiological parameters to assess HA: cardiovascular changes (i.e., HR), temperatures (i.e., *T*_re_) and SR.

**Table 3:** Overview of the studies included in the review

Study	Heat acclimation						Heat stress test			
	Days	Temperature (°C)	Relative humidity (%)	Intensity	Duration (min)	Exercise modality	Temperature (°C)	Relative humidity (%)	Intensity	Duration (min)
Adam et al. [99]	12	Hot		116 W/m^2^	120	Load carrying	36	76	116 W/m^2^	120
Ashley et al. [102]	6	50	20	40% VO_2max_	< 120	Walking	50	20	40% VO_2max_	< 120
Bean and Eichna [78]	7–21	49	15–22		180	Load carrying	49	15-22		180
Daanen et al. [93]	9 + 3^a^	35/41	29/33	45% VO_2max_ + max	120	Cycling	35	29	45% VO_2max_ + max	120
Flouris et al. [103]	14	40	20	50% VO_2max_	90	Cycling	35	16	300,350,400 W/m^2^	90
Garrett et al. [34]	5	39.5	60	*T*_re_ 38.5	90	Cycling	35	60	40% PPO + max	90
Gill and Sleivert [23]	10	38	70	70% VO_2max_	30	Rowing	38	70	70% VO_2max_	30
Henschel et al. [98]	2	43-49	20–25	7 times BMR	120	Walking	43–49	20–25	7 times BMR	120
Lee et al. [107]	Permanent	27	81	Passive	Permanent	Passive	24	40	Iontophoresis	5
Lee et al. [108]	Permanent	Hot		Passive	Permanent	Passive	0.1 °C/s		Local *T*_sk_ change	
Lind and Bass [39]	9	49	17	350 W EE	50–100	Walking	49	17	350 W EE	100
Neal et al. [105]	11	39	56	*T*_re_ 38.5	90	Cycling	39	53	35% PPO	60
Pandolf et al. [91]	9	49	20		110	Walking	49	20		110
Pichan et al. [111]	8	45	30	465 W EE	100		45	30	465 W EE	100
Poirier et al. [42]	14	40	20	50% VO_2max_	90	Cycling	35	16	300,350,400 W/m^2^	
Saat et al. [101]	14	Hot		60% VO_2max_	60	Cycling	31	70	60% VO_2max_	60
Stephens and Hoag [110]	10	37–41	35–75	378 W EE	100	Walking	37–41	35–75	378 W EE	100
Weller et al. [75]	10	46	18	*T*_re_ 38.5	100	Inclined walking	46	18	*T*_re_ 38.5	100
Wijayanto et al. [109]	Permanent	28	80	Passive	Permanent	Passive	28	50	Leg in 42 °C water	60
Williams et al. [92]	4^b^	34	80	340 W EE	< 300	Step test	34	80	340 W EE	< 300
Wyndham et al. [41]	12	Hot mine				Work	Hot mine		At rest	

*BMR* basal metabolic rate, *EE* energy expenditure, *PPO* peak power output, *T*_*re*_ rectal temperature, *max* maximal exercise until exhaustion, *T*_*sk*_ mean skin temperature, *VO*_*2max*_ maximal oxygen uptake, *WBGT* wet bulb globe temperature

^a^9 days at 26° C WBGT and 3 days at 32° C WBGT

^b^4 days of controlled heat acclimation after 12 days of uncontrolled exposure to a warm environment

**Table 4:** Overview of the variables measured in the studies included in the review

Study	HR	*T*_re_	*T*_es_	*T*_or_	*T*_sk_	SR	RPE	TS	TC	HRV	VO_2_	Cal	PV	Blood^a^	Lactate	SW	Performance
Adam et al. [99]	×	×				×											
Ashley et al. [102]	×	×															×
Bean and Eichna [78]	×	×															
Daanen et al. [93]	×	×			×	×	×	×	×								×
Flouris et al. [103]	×	×	×							×							
Garrett et al. [34]	×	×											×	×			×
Gill and Sleivert [23]	×	×			×	×	×										
Henschel et al. [98]	×	×				×											
Lee et al. [107]						×											
Lee et al. [108]					×												
Lind and Bass [39]	×	×				×											
Neal et al. [105]	×	×			×	×	×	×	×				×	×			
Pandolf et al. [91]	×	×			×	×	×	×									
Pichan et al. [111]	×			×		×										×	
Poirier et al. [42]	×	×	×			×						×					
Saat et al. [101]	×	×				×	×	×							×		
Stephens and Hoag [110]	×	×			×	×											
Weller et al. [75]	×	×			×	×	×	×			×						
Wijayanto et al. [109]	×	×			×	×											
Williams et al. [92]	×	×				×											
Wyndham et al. [41]				×													

*Cal* whole-body calorimetry using the Snellen calorimeter, *HR* heart rate (bpm), *HRV* heart rate variability, *lactate* plasma lactate (mmol/L), *performance* tolerance time or time to exhaustion, *PV* plasma volume (L), *RPE* rating of perceived exertion, *SR* sweat rate (L/h), *SW* sweat analysis for sodium and potassium concentrations, *TC* thermal comfort, *T*es esophageal temperature (°C), *T*_*or*_ oral temperature (°C), *T*_*re*_ rectal temperature (°C), *TS* thermal sensation, *T*_*sk*_ mean skin temperature (°C), *VO*_*2*_ oxygen uptake (L/min)

^a^Blood analysis including fluid regulatory and stress hormones, total protein, and albumin and plasma sodium concentration [34], hemoglobin concentration, hematocrit, aldosterone concentration, and concentration of heat shock protein (HSP70) [105]

#### Cardiovascular Changes

**Fig. 6:**
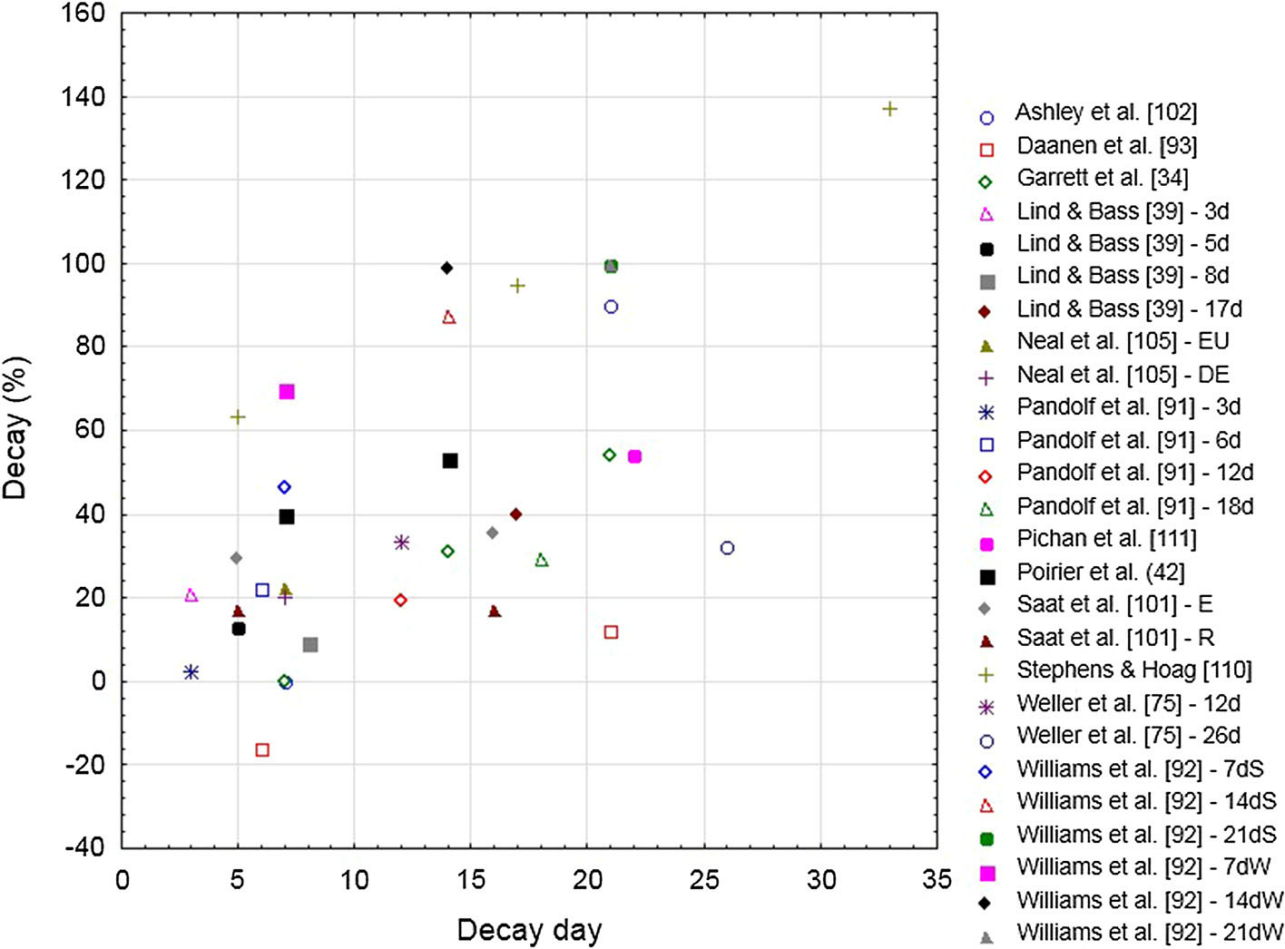
Decay (%) in the heart rate adaptation following heat acclimation relative to the number of decay days (i.e., without heat exposure) for selected studies. Studies that appear more than once in the figure are longitudinal studies in which heat acclimation decay was tested multiple times in a single heat acclimation group. *d* days after heat acclimation, *DE* dehydration, *E* exercise, *EU* euhydration, *R* rest, *S* summer, *W* winter

HR is highly influenced by HA and is affected by changes in skin blood flow, increasing to compensate for the reduced blood volume in the central circulation [15]. The relationship between the decay in HR adaptations during exercise and the number of HAD days is shown in Fig. 6.

##### Transverse (Cross-Sectional) Studies

Weller et al. [75] evaluated two groups of eight participants, with one group returning to re-acclimate for 5 days after a period of 12 days without heat stress and the other group for 7 days after 26 days without heat exposure. They observed that the HA-induced reduction in HR decayed by a mean of ~ 30% in both groups (Fig. 6). Pandolf et al. [91] heat-acclimated four groups of six soldiers who returned for 2 days of HRA 3, 6, 12, and 18 days after an initial 9-day HA regimen. They observed that fit soldiers (i.e., high VO_2max_) showed rapid HA and a slow HAD in the HR adaptation (2–29%), which on days 12 and 18 of HAD remained similar to that of day 9. Pichan et al. [111] investigated HAD on one occasion, 21 days after HA. Twelve soldiers were heat acclimatized for 8 days and thereafter exposed to a cold environment (10 °C) for 4 h/day during 3 weeks. They observed a mean HAD in HR of 54%, which was greater than that of Pandolf et al. [91], perhaps due to tropical heat exposure and then to the cold, which may have accentuated the rate of decay. Williams et al. [92] formed three groups of 16–18 Africans who were heat acclimatized for 12 days and acclimated for an additional 4 days in summer and winter. Participants were re-tested either 7, 14, or 21 days following each respective HA regimen. The authors demonstrated that HR adapted most quickly compared with *T*_re_ and SR during HA, but that it also returned to previous values most quickly during HAD. Interestingly, HR returned more quickly to the unacclimatized state during the winter condition than in summer, with averaged losses of acclimatization of 70 and 46% after 1 week. A 99–100% loss of adaptation was noted in both seasons 3 weeks post-acclimatization (Fig. 6).

Following a 9-day HA regimen in four groups, Lind and Bass [39] examined HR responses in two to three participants per group upon returning for 5 days of HRA on days 3, 5, 8, or 17. Most adaptations were still present after 3–8 days, but not after 17 days. Similarly, Bean and Eichna [78] observed in seven participants that the adaptations in HR were well-maintained for 1 week but lost by most participants after a month. These adaptations, however, were acquired over varying HA periods (e.g., 1–3 weeks), and as such must be interpreted with caution. Henschel et al. [98] heat acclimated 24 male participants for 2 days only and examined HAD in eight men after 1 week, five after 2 weeks, seven after 3 weeks, and four after 4 weeks. A reduced HR was observed for the groups that returned 1–3 weeks after HA, but not for the group that returned 4 weeks later. Since 2 days of HA is considered too short to elicit significant changes, these values were not included in Fig. 6.

##### Longitudinal Studies

Flouris et al. [103] and Poirier et al. [42] examined different variables from the same experiment (Table 3). Mean HR was 176 bpm prior to heat exposure and 161 bpm after complete HA. They observed a mean HR of 167 bpm or a mean HAD of 40% in HR after heavy exercise (400 W/m^2^) 1 week after HA (Fig. 6), which was not significantly different from the acclimated value [42]. A week later the mean HR was 169 bpm or mean decay was 53%, but was still not significantly different from complete HA. Flouris et al. [103] reported that HA led to parasympathetic system dominance. Thus, it seems that HA modifies the way blood vessels may be controlled (i.e., greater vasodilation) and that HAD may also be the result of altered blood vessel control with a gradual increase in peripheral vasoconstriction.

A recent study by Ashley et al. [102] examined 10 days of HA in six participants returning for HAD testing either 1, 3, or 5 weeks after HA. One week after HA, the HR response showed no decay and after week 3 all adaptations were gone, with the authors suggesting that the loss of acclimation occurred in the first 2 weeks of absence from the heat. Similarly, Garrett et al. [34] induced HA over 5 days using controlled hyperthermia and observed that adaptations in HR persisted for 1 week but not 2 weeks. Notwithstanding, only a mean decay of 31% in HR was observed after 2 weeks and a mean decay of 54% after 3 weeks without heat exposure. Daanen et al. [93] also observed a slow decay. In this study, 15 males performed maximal exercise for 9 days at a WBGT of 26 °C and an additional 3 days at 32 °C. HR at the end of exercise decreased from an average of 154 bpm on the first day of the HA period to an average of 129 bpm on day 9. Three days after full HA, HR still averaged 129 bpm at the end of exercise with a slow decay to 132 bpm 3 weeks after HA.

Saat et al. [101] induced HA by exposing participants to 14 days of daily exercise (1 h) on a cycle ergometer at 60% VO_2max_ in a hot/humid environment. Following HA, the investigators exposed 16 males to a relatively cool climate (18 °C) for 1 h in the morning and 1 h in the afternoon. Eight males had to perform exercise in the morning (60% VO_2max_) while the other eight did not exercise. The mean decay in HR 5 and 16 days after HA was 29–35% for the group exercising in the cold and 17% for the group not exercising in the cold. The unexpected finding that exercising in cool conditions enhanced decay rather than attenuated it could not be explained by the authors. The decay was similar 5 and 16 days after HA, showing that the number of decay days played a minor role in their results. Finally, Stephens and Hoag [110] examined HAD in six females returning 5, 17, and 33 days after a HA regimen in order to re-induce complete HRA over 4–6 days. They reported data from day 4 of HA onwards and, as such, HAD cannot be quantified in the standard manner (see Fig. 2; parameter A is missing). Therefore, HR prior to HA was estimated using extrapolation to a value of 155 bpm. The authors reported that after 5 days of HAD, HR had decreased to values similar to those on day 4 of HA, which corresponded to a considerable mean decay of 63%. The decay in HR was higher after 17 and 33 days of HAD (Fig. 6).

##### Regression Analysis

Twelve studies were included in the stepwise multiple regression analysis [34, 39, 42, 75, 91, 93, 101–103, 105, 110, 111]. When the percentage decay in HR was used as the dependent variable and the number of HA and HAD days, WBGT, and daily exposure duration as independent variables, the following regression equation (Eq. 2) resulted after stepwise elimination of non-significant variables, with a correlation coefficient (*r*) of 0.60 (*P* < 0.001):

2 $$ {\text{Decay in HR }}\left( \% \right) = 3.6 \, \left( {P = 0.69} \right) + 2.3 \, \left( {P < 0.001} \right) \times {\text{no}} . {\text{ of decay days }}\left( {n = 29} \right). $$

This equation indicates that the number of HA days and the duration/intensity of daily heat exposure do not affect the decay. It is likely that HA was sufficient to induce stable adaptations in HR. The adaptations were slowly lost at a rate of 2.3% per decay day.

#### Core Temperature Changes

**Fig. 7:**
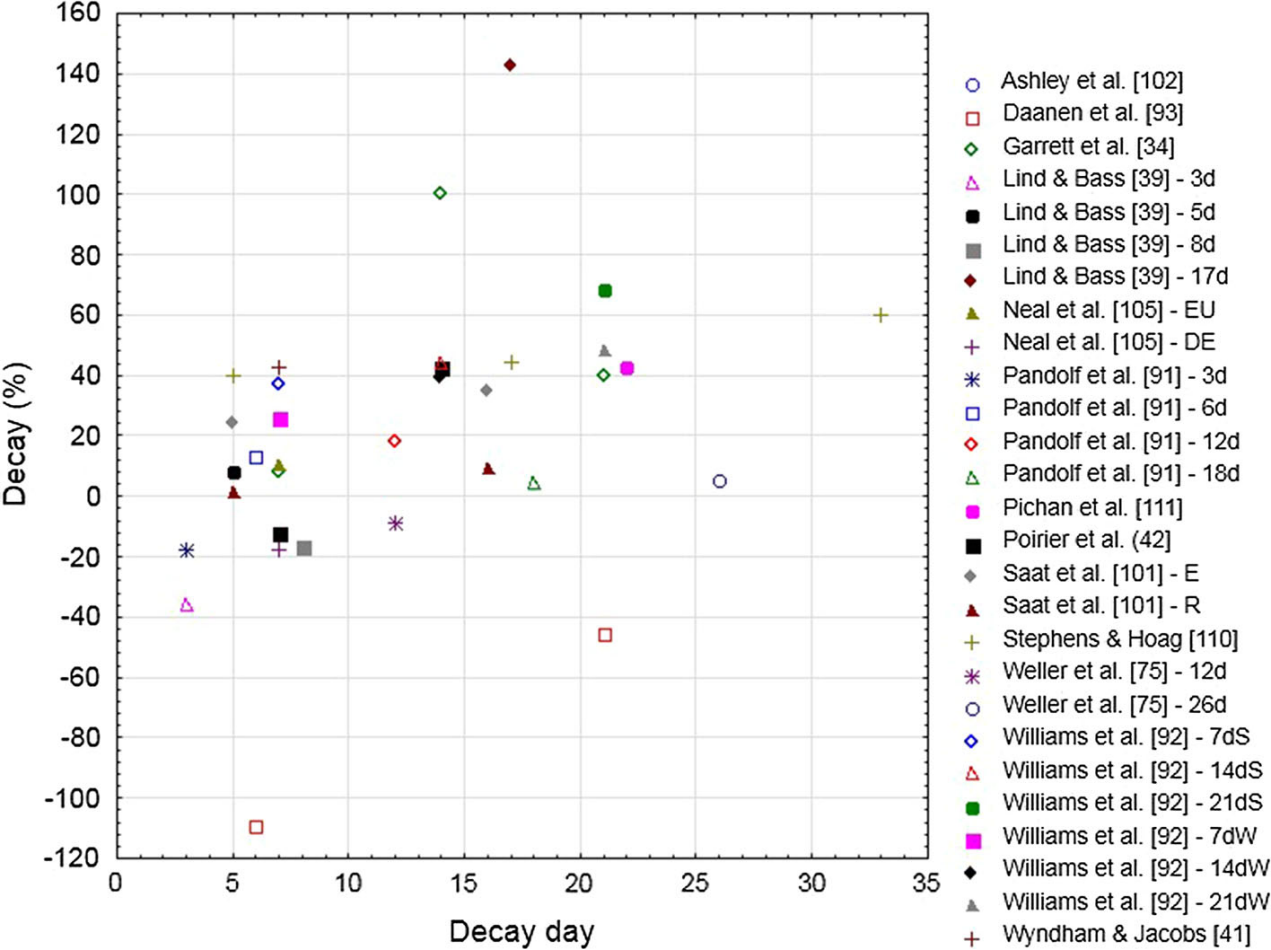
Decay (%) in the body core temperature adaptation following heat acclimation relative to the number of decay days (i.e., without heat exposure) for selected studies. Studies that appear more than once in the figure are longitudinal studies in which heat acclimation decay was tested multiple times in a single heat acclimation group. *d* days after heat acclimation, *DE* dehydration, *E* exercise, *EU* euhydration, *R* rest, *S* summer, *W* winter

*T*_c_ at rest and at the end of exercise reflects the autonomic thermoregulatory adaptations that occur during HA. *T*_c_ prior to heat exposure was investigated in four studies [34, 75, 93, 111] and mean HAD ranged from − 246% [93] to + 180% [34] due to small changes during HA, thus leading to inconclusive results. The − 246% value was the result of a large decrease (0.3 °C) in resting *T*_re_ after but not during HA, due to a reduction in the ability to recover from the heat during the HA period. Figure 7 shows the relationship between the decay in *T*_c_ at the end of exercise and the number of HAD days.

Weller et al. [75] observed that the adaptations in post-exercise *T*_re_ were persistent with a mean decay of only 5% after 26 days (Fig. 7). Poirier et al. [42], however, observed a mean decay in *T*_re_ of 43% after 2 weeks [42], as did Pichan et al. [111] (43%) after 3 weeks, which illustrates the wide scatter of results. Pandolf et al. [91] showed minimal HAD (<  20%) over 18 days, while Williams et al. [92] observed a large mean decay of 69% in *T*_c_ adaptations after 3 weeks. Williams et al. [92] observed that fit soldiers showed faster HA, slower HAD, and faster HRA for *T*_re_ at the end of exercise. Ashley et al. [102] failed to observed HA adaptations in *T*_c_, and as such HAD cannot be calculated. A likely reason for this absence of changes in *T*_c_ is that the exercise period increased during the HA period from 102 to 115 min. In particular, during the initial days of HA, several participants had to stop because of fatigue before the 2 h of walking were attained. In the study by Daanen et al. [93], the extreme heat strain during exercise and lack of cool facilities during recovery are a likely explanation for the observation that minimal physiological changes occurred during the 12 days of HA. In contrast, the HAD period seems to have been restful enough to induce the HA adaptations. A 0.5 °C lower mean *T*_re_ was observed at the end of exercise 3 days after HA, relative to pre-acclimation temperature. This seems to indicate that adequate recovery in cool conditions is required between HA sessions in extreme conditions to allow adaptation. Although it appears that delayed adaptations develop following a strenuous HA protocol, this situation is likely undesirable.

Garrett et al. [34] observed that adaptations in *T*_re_ persisted for 1 week (8% mean decay, or 0.02 °C) but not two (100% decay), likely in response to the short HA period utilized. Interestingly, Saat et al. [101] observed differences between exercising and non-exercising groups in *T*_re_, which decayed at a faster rate after performing exercise in the post-HA period (average 24%) relative to remaining inactive (average 2%) 5 days after complete HA. Since exercise increases *T*_c_ and induces sweating, even in cool conditions, this represents an unexpected result as exercising in cool conditions enhanced decay rather than attenuated it. In fact, Pandolf et al. [91] noted that aerobic fitness and regular exercise contribute to retaining the benefits of HA. The hypothesis of Saat et al. [101] that heat strain during exercise in the cold may have led to enhanced heat loss is counteracted by the fact that there is no need for enhanced heat loss in the cold when exercising at a given workload. Moreover, it has been shown that repeated exposure to cold does not change heat production and heat loss during exercise [112].

Stephens and Hoag [110] observed a mean decay in *T*_re_ of 40% after 5 days, 44% after 17 days, and 60% after 33 days. In contrast, Lind and Bass [39] found that *T*_re_ adaptations persisted for 3–8 days, but not for 17 days, which is somewhat similar to the results of Adam et al. [99], who observed a lower *T*_c_ after 6, but not 28, days of HAD. Wyndham and Jacobs [100] observed a considerable mean drop of 0.4 °C in oral temperature after 6 days of HAD, suggesting that the decay in oral temperature may be faster than for *T*_re_. This is in line with the notion that body temperature is a specific expression of the local heat balance in particular locations of the body [113], and thus may involve different thermoregulatory rates of heat loss and gain.

Whilst considerable differences exist between study designs and results (Table 3), a general picture emerges in that the decay in *T*_c_ is slower than for HR, and that the adaptations lowering body temperature persist to various extents over several weeks after HA. The two studies reporting relatively fast decay in *T*_c_ are either poorly quantified [99] or based on a very small number of participants [39].

##### Regression Analysis

Eleven studies were included in the stepwise multiple regression analysis for *T*_c_ decay [34, 39, 42, 75, 91, 93, 101, 103, 105, 110, 111]. When the percentage decay in *T*_c_ was used as the dependent variable and the number of HA and HAD days, WBGT, and daily exposure duration as independent variables, the following regression equation (Eq. 3) resulted after stepwise elimination of non-significant variables, with a correlation coefficient equal to 0.69 (*P* = 0.002):

3 $$ {\text{Decay in }}T_{\text{c}} \, \left( \% \right) \, = \, {-}126 \, \left( {P = 0.11} \right) \, + \, 2.6 \, \left( {P = 0.007} \right) \, \times {\text{ no}} . {\text{ of decay days }}{-} \, 1.2 \, \left( {P = 0.007} \right) \, \times {\text{ duration }} + \, 6.8 \, \left( {P = 0.006} \right) \, \times {\text{ WBGT }}\left( {n = 27} \right) . $$

This indicates that number of HA days does not affect the decay. It is likely that HA was sufficient to elicit relatively stable adaptations in *T*_c_. The adaptations were slowly lost at a rate of 2.6% per decay day. Of note, increasing the duration of daily heat exposure and reducing the intensity of heat exposure during daily HA reduced the rate of decay. For instance, extending daily exposure from 60 to 90 min at a WBGT of 32 °C reduced HAD in *T*_c_ from 45 to 9%.

#### Sweat Rate

**Fig. 8:**
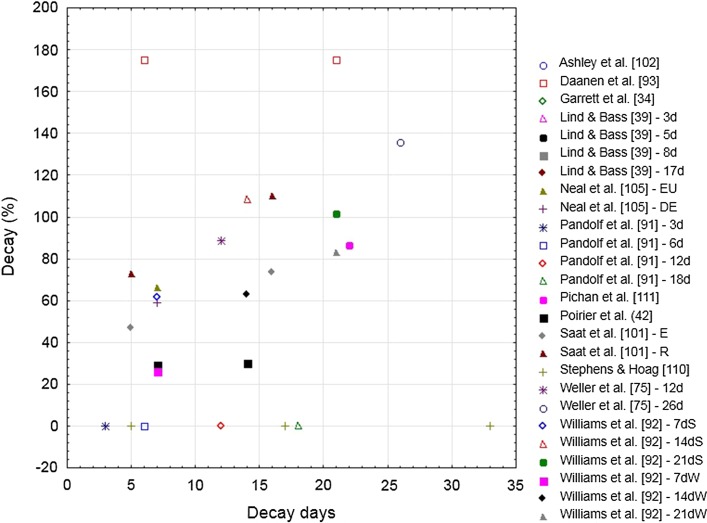
Decay (%) in the sweat rate adaptation following heat acclimation relative to the number of decay days (i.e., without heat exposure) for selected studies. Studies that appear more than once in the figure are longitudinal studies in which heat acclimation decay was tested multiple times in a single heat acclimation group. *d* days after heat acclimation, *DE* dehydration, *E* exercise, *EU* euhydration, *R* rest, *S* summer, *W* winter

An increase in SR enhances the evaporative power and cooling capabilities of the human body in environmental conditions conducive to evaporation. This adaptation represents an important pathway via which thermoregulation is enhanced under heat stress. Figure 8 shows the relationship between the percentage change in SR and the number of HAD days.

Calculation of HAD is only possible when changes are observed during HA. Two studies found no changes in SR during HA [91, 110]. In Fig. 8 these values are shown as 0% decay and the datapoints were excluded from the regression analysis. For the other studies, the range in which SR increased during HA averaged 21%, with a considerable standard deviation of 13%. The increase in SR during HA may be influenced by many factors, including training status and partial HA prior to undertaking a HA regimen; the age, sex, morphology and ethnicity of participants; the exercise intensity and duration of HA; and the type of exercise employed during HA. Therefore, no clear factor can be identified that may explain why the studies by Pandolf et al. [91] and Stephens and Hoag [110] did not observe an increase in SR during HA.

Weller et al. [75] showed that SR adaptations were completely lost after a decay period of 26 days. This is in line with Adam et al. [99] who observed complete decay after 28 days. However, this article presented no data—only levels of significance—so it could not be included in the analysis. Most studies reporting SR observed a fast decay ranging from 26 to 175% following 5–7 days of HAD, and 63–175% 2 weeks or more after HA [92, 93, 101, 111]. In the study by Daanen et al. [93], mean SR increased only slightly from 1.00 to 1.04 L/h. This small increase forms the reference for the decay calculation and therefore leads to a value of 175% for a SR of 0.97 L/h after HAD. Poirier et al. [42] observed a non-significant mean decay in SR of 30% 1 and 2 weeks after HA. Similarly, Henschel et al. [98] observed no changes in SR between the values at the end of HA and those after 1, 2, 3, or 4 weeks of HAD. However, their HA period was only 2 days, which is likely much too short to induce changes in SR. Moreover, SR varies considerably from day to day within participants [39]. These results suggest that adaptations in SR do not systematically occur during HA, making HAD hard to quantify.

##### Regression Analysis

Seven studies were included in the stepwise multiple regression analysis [42, 75, 93, 101, 103, 105, 111]. When the percentage decay in SR was used as the dependent variable and the number of HA and HAD days, WBGT, and daily heat exposure duration as independent variables, the following regression equation (Eq. 4) resulted after stepwise elimination of non-significant variables, with a correlation coefficient of 0.82 (*P* = 0.004):

4 $$ {\text{Decay in SR }}\left( \% \right) \, = \, 964 \, \left( {P < 0.001} \right) \, {-} \, 27.7 \, \left( {P = 0.002} \right) \, \times {\text{ no}} . {\text{ of HA days }}{-} \, 18.2 \, \left( {P = 0.002} \right) \, \times {\text{ WBGT }}\left( {n = 13} \right). $$

Unfortunately, the number of studies included is small since several studies had to be excluded (Sect. 3.1.1). Additional research is thus required to draw meaningful conclusions. Notwithstanding, the limited dataset showed a significant dependency in SR decay on the number of HA days. A longer period of HA led to more sustained adaptations, which is in line with earlier observations that it takes longer to induce adaptations in SR, relative to HR or *T*_c_ [12]. Moreover, the regression equation indicated that a high WBGT during HA related to a reduced rate of decay. It is likely that challenging conditions form an important stimulus for enhancing SR.

#### Mean Skin Temperature

The studies that investigated the decay in end-exercise *T*_sk_ [75, 93, 105, 110] observed a consistent mean decrease of 0.9 °C (range 0.5–1.2 °C) during HA but conflicting results on decay. Weller et al. [75] found a slow decay of 8% after 12 days and 56% after 26 days. Neal et al. [105] observed minor changes in Tsk after a decay period of 7 days. In contrast, Stephens and Hoag [110] and Daanen et al. [93] observed a fast decay of 67% after 5 days and 57% after 6 days, respectively. More research is needed to draw solid conclusions.

#### Performance

Three papers investigated changes in performance during HAD [34, 93, 102]. One study indicated that tolerance time remained unchanged between the end of HA (115 min) and 3 weeks of HAD (116 min) [102]. However, tolerance time in this study was not very sensitive: most participants completed the 120 min at 40% VO_2max_ treadmill work with ease and only a few met the stop criteria based on *T*_c_, HR, or voluntary cessation. Similar values were observed in another study where tolerance time was 46.5 min after 9 days of HA and 46.4 min 1 week later when performing exercise to exhaustion [93]. Garrett et al. [34] evaluated mean performance duration in a maximal ramp protocol, which lasted 12.4 min prior to HA, 14.2 min after HA, 13.7 min after 1 week of HAD, and 12.7 min after 2 weeks of HAD. Based on these limited data, improvements in performance following HA seem to persist for 1–2 weeks in the HAD period.

#### Subjective Ratings

Ratings of perceived exertion (RPE) and TS were investigated in five studies [75, 91, 93, 101, 105]. All studies observed a reduction in RPE and TS during HA. Pandolf et al. [91] did not quantify RPE and TS during decay, whereas Daanen et al. [93] observed that RPE and TS after 60 min of cycling at 45% VO_2max_ remained unchanged between the end of HA and 7 or 18 days later. Saat et al. [101] showed a considerable reduction in mean RPE from 16.7 to 14.4 (on the 6–20 scale) during 60 min cycling at 60% VO_2max_ in the HA period, and a small decay a month later (RPE of 13.7). They also noted that TS remained unchanged. Weller et al. [75] observed unchanged RPE and TS values 12 or 26 days after HA during 60 min of inclined treadmill walking. The same was found by Neal et al. [105] 7 days after HA. Thus, it seems that RPE and TS increase only slightly during HAD, even over the time span of a month. This observation is based on limited data, however, and on exercise varying considerably in intensity.

#### Plasma Volume

Plasma volume changes were investigated by Garrett et al. [34] who observed no changes during and after HA. This was in contrast to the finding of Neal et al. [105] who showed an increase in plasma volume during HA but no significant decrease after 7 days of decay.

### Heat Re-Acclimation Studies

Nine studies investigated HRA, with Pandolf et al. [91], Henschel et al. [98], and Wyndham and Jacobs [100] using 2 days and other studies at least 3 days to re-acclimate [39, 75, 101, 102, 110, 111]. Transversal and longitudinal studies were included. Ashley et al. [102] described two studies: the one described previously in the Longitudinal Studies section and a dedicated HRA study in which four participants returned to the laboratory 2 weeks after HA and four participants 4 weeks after HA for HRA until *T*_re_ was stable for 3 consecutive days. The concept of HRA is similar to HA, but since some physiological adaptations may be retained to various extents if decay is not complete, the expectation is that HRA may be faster than HA. Even if HAD is fast, the possibility that the previous physiological adaptations may influence HRA should not be excluded.

#### Cardiovascular Changes

All studies show that HRA is much faster at lowering absolute HR than HA [75, 91, 101, 102, 110, 111]. The main reason is likely that part of the adaptation is retained (C higher than A in Fig. 3); however, HRA also increases the rate at which HR is reduced. For example, Stephens and Hoag [110] showed a mean decline in HR of about 3 bpm/day during HA, whereas a decrease of 4 bpm/day was noted during the first HRA period (4 days) in female participants. This was followed by a mean increased rate of 7 bpm/day during the second (4 days) and third (6 days) HRA periods. The authors fitted the data with a regression line for end of exercise HR and proposed the following formula (Eq. 5):

5 $$ {\text{HR }} = \, 148 \, + \, 0.76 \, \left( {\text{decay days}} \right) \, {-} \, 5.86 \, \left( {\text{re - acclimation days}} \right) \, \left( {r = 0.86} \right). $$

A striking finding was that HRA was about eight times stronger than the effect of HAD. In other words, a month of HAD could be compensated for by only 4 days of HRA. It has also been shown that 2 days were sufficient for HR to return to HA levels after 12 days of HAD, whereas 4 days were required to compensate for 26 days of HAD [75]. Although Pandolf et al. [91] only studied 2 days of HRA, considerable differences of 1, 5, 8, and 11 bpm were noted in HR between the first and second day of HRA starting 3, 6, 12, and 18 days after HA, respectively. Others have shown similar results with faster adaptations for HR during HRA than HA, with HR at the end of exercise being identical after 3 days of HRA and 8 days of HA [111].

Interestingly, it has been shown in certain studies that HRA induces greater adaptations than HA [39, 101]. Indeed, at the end of a 10-day HRA protocol, HR was lower than at the end of a 14-day HA regimen [101]. This suggests that a long period of HRA may enhance adaptations beyond those occurring during HA (D became lower than B in Fig. 3). Another study observed changes in both HR and *T*_re_ after 5 days of HRA that surpassed those of HA, but in this study only 11 participants out of 16 successfully completed the experiment [39]. Although it cannot be excluded that HA was incomplete in both studies due to the relatively short daily exposures of 60 min [101] and 50–100 min [39], these studies highlight the potential for prolonged HRA to induce greater adaptations compared with incomplete (i.e., shorter) HRA [91], or HRA of a fixed timeframe (5–7 days) [75, 111]. Notwithstanding, HRA in all studies was considerably faster than HA for inducing adaptations in HR.

#### Core Temperature Changes

Given that the decay in *T*_c_ after HA is relatively moderate, adaptations generally remain when HRA is undertaken, thus allowing for rapid HRA. For example, only 1 day of HRA was necessary for *T*_re_ to return to HA levels, even after 26 days of HAD in the Weller et al. [75] study. The extremely fast re-acclimation was related to a minimal decay. Wyndham and Jacobs [100] also showed that mean oral temperature decreased from 37.6 °C during the first day of HRA to 37.3 °C on the second day—a value similar to the end of HA. Pichan et al. [111] observed full recovery in 3 days, whereas Lind and Bass [39] observed a reduction in mean *T*_re_ during 5 days of HRA of only 0.05 °C for two participants who started re-acclimation after 3 days of HAD. They also noted a 0.7 °C decrease in mean *T*_re_ for the three participants undertaking HRA 17 days after HA. Accordingly, with a longer HAD period a greater magnitude of adaptation is lost and, consequently, more pronounced gains may occur during HRA. A long HRA period may be beneficial as reflected by a lower *T*_re_ at the end of a 10-day HRA protocol relative to the end of the 14-day HA regimen [101].

Two studies re-acclimated until either *T*_re_ reached a plateau for 3 days [102] or until a similar status to that at the end of HA was achieved [110]. On average it took 6.1 days to reach a plateau in *T*_re_, whilst 3.8 days were required to re-acclimate after 2 weeks of decay and 5.3 days after 4 weeks of decay [102]. Stephens and Hoag [110] modelled HAD and HRA in *T*_c_ at the end of exercise as follows (Eq. 6):

6 $$ T_{\text{re}} \, = \, 38.3 \, + \, 0.006 \, \left( {\text{decay days}} \right) \, {-} \, 0.07 \, \left( {\text{re - acclimation days}} \right) \, \left( {r = 0.73} \right). $$

Stephens and Hoag [110] showed that HRA was about 12 times stronger than the effect of HAD. It can be concluded that HRA is fast for *T*_c_, mainly because the adaptations in *T*_c_ are still largely present at the moment of HRA, but also to some extent in relation to rapid daily adaptations occurring during HRA, as observed with HR.

#### Sweat Rate

Two studies observed only minimal changes in mean SR during HA and HRA [91, 110]. Pichan et al. [111] observed a small improvement over 5 days from 0.56 to 0.59 L/h (end HA value 0.63 L/h). The moderate exercise intensity of this study may explain these low SR. Similarly, Weller et al. [75] found a mean increase from 0.93 to 1.03 L/h (end HA 1.1 L/h) over 5 days. In this study, 2 days were sufficient for SR to return to HA levels after 12 days of HAD, whereas 4 days were required to compensate for 26 days of HAD. An average increase of 0.97–1.11 L/h (end HA 1.16 L/h) over the course of 12 days of HRA has also been observed [101]. Finally, Henschel et al. [98] observed non-consistent changes when comparing the second day of HRA to the first day, but this may also have been due to the short HA period of 2 days. Hence, the adaptations in SR during HA and HRA do not seem to differ and show a small increase over days, but limited data are available to substantiate this conclusion.

### Other Types of Studies

#### Population Studies

Three studies compared the physiological differences between a population living in a cold climate for several years and a population that lived in a tropical climate which then moved to the colder region [107–109]. The population indigenous to the tropics was considered to be adapted to heat. The physiological changes in this population after living in the relatively colder climate were considered to represent the decay in prolonged/chronic HA, more specifically long-term decay. The main goal was to investigate the changes in physiological parameters occurring in the tropical population over several months as their responses became comparable to those of the native (i.e., Japanese) population. The sweat response to acetylcholine iontophoresis was generally suppressed in the tropical population and it was observed that it increased after residing in the colder climate, doubling after 50 months of residence [107]. This suggests that individuals moving from a tropical climate lose part of their HA status after living for an extended time period in a colder climate. In line with this, the onset of sweating occurred earlier in the tropical population with longer residence [107, 109]. If we assume that the tropical population was heat adapted upon moving to the colder climate, then a slow increase in mean SR can be observed during HDA of about 1.1 [109] to 1.7% per month [107]. This long-term adjustment opposes that noted in short-term decay, with SR typically decreasing. Lee et al. [107] suggested calling these adaptations “deacclimation”, whereby SR of the tropical population residing in the cold climate gradually increases to values similar to those of the population already living in the cold. The physiological process underlying these changes seem unrelated to alterations in cutaneous sensitivity since it does not change with residence time [109], but may be related to alterations in sympathetic/parasympathetic activation, similar to those observed by Flouris et al. [103] during HA. In addition, observations on populations may be biased since natives of hot countries are not as acclimatized as people going through a dedicated HA protocol [114].

#### Interval Studies

The study by Givoni and Goldman [115] is considered an important one regarding HAD [110], but is not included in this review as HAD and HRA were not the focus of research. For example, the authors combined data from five partly published studies in which HA was not performed every day, with some days omitted (e.g., the weekends). When a particular data point was missing (e.g., *T*_re_), a correction factor was used whereby half of the change in *T*_re_ from the previous day of HA was calculated. This yielded a reasonable exponential fit for HA, which was in line with the later work of Gill and Sleivert [23] showing that daily HA was far more successful than acclimation every other day (i.e., intermittent HA). Daily exposure to heat in that study led to a mean end-exercise *T*_re_ of 38.0 °C at the end of HA, while intermittent exposure led to a final mean *T*_re_ of 38.5 °C; mean end-exercise HR values were 165 and 175 bpm, respectively, whilst final RPE stabilized at 11 for daily exposures and 16 for intermittent HA [23]. Accordingly, it appears that intermittent HA leads to suboptimal adaptations.

## Discussion

There is a long history of HA studies and several recent reviews confirm its impact on performance in the heat [12, 14, 15]. When effectively performed and monitored, HA may be integrated successfully in training programs. The numerous well-documented studies on HA are in contrast with the limited number of studies available on HAD and HRA. These studies mainly focus on adaptations related to HR, *T*_c_, and SR. The rate of decay in adaptations varies considerably between studies, probably due to the large number of confounders, such as duration and type of HA, acclimation status of the participants prior to HA, and physical activity (i.e., exercise), along with ambient conditions during decay.

The stepwise multiple regression showed a decay in HR and *T*_c_ of about 2.5% for every day of decay. The number of HA days did not contribute to the decay in adaptations, suggesting that at least 5 days of HA is sufficient to evoke relatively stable adaptations. This is in line with the main findings outlined in a recent review on HA [12]. It appears that the number of HA days affects the decay in SR, with longer HA periods leading to less decay (i.e., more sustainable adaptations). Therefore, it can be suggested that protracted HA regimens are beneficial for adaptations in SR. It was also shown that longer daily heat exposure has a considerable benefit for adaptations in *T*_c_. Higher daily WBGT conditions are of benefit for adaptations in SR, but seem to be disadvantageous for adaptations in *T*_c_. Since adaptations in *T*_c_ can be enhanced by extending daily exposure, maintaining WBGT at a sufficiently high level (e.g., >  30 °C) to generate stable adaptations in SR may be advisable.

### Limitations

Decay in end-exercise HR and body *T*_c_ is strongly dependent on the number of HA days and to a lesser extent on the number of HAD days. For SR, the regression is not significant due to large variations. Linear regression was used for the analysis as it is unlikely that other regression techniques would have resulted in better fits (Figs. 6, 7 and 8). The variation in HAD is also dependent on inconclusive changes in SR during HA. The ambient conditions during decay were rarely specified or controlled for in the available studies. Only two studies clearly specified the conditions during decay [101, 111]. In the other studies, non-exposure to heat was generally the only mentioned factor. Williams et al. [92] compared the decay in summer with winter, and observed a slightly faster decay in winter for *T*_re_. The observation of Saat et al. [101] that exercise in the cold period after HA leads to a faster decay is somewhat counterintuitive as aerobic fitness and regular exercise have been suggested to be critical during HAD in providing stimulus for sustaining adaptation [91].

During HRA, ambient conditions in the selected studies varied from ~ 30 to ~ 50 °C, with relative humidity ranging from 20 to 80% (Table 3). Exposure duration also varied considerably from 1 h [101] to almost 5 h [92]. The 1 h exposure does meet the timeframe proposed by Houmard et al. [77], but not that suggested by Taylor [13]. The participants in these studies were mainly fit young males with mean VO_2max_ values >  50 mL/min/kg (Table 1), which renders extrapolation of the results to less fit and female populations difficult. There is a considerable variation in the acclimation status of the participants prior to the HA protocols, which may have affected the changes during HA. Some studies started in summer time, others in the winter period, which may have affected the results [92]. It is uncertain if the HA protocols employed in the investigated studies led to complete HA as some studies had a limited number of HA days (e.g., Garrett et al. [34]), while others had relatively short daily heat exposure durations (e.g., Saat et al. [101]).

This review demonstrates that HR, *T*_c_, and SR follow different patterns of induction, decay, and re-induction, even though these variables are intimately linked. HA leads to a variable increase in plasma volume (see Périard et al. [15] for review). Unfortunately, only two studies investigated plasma volume changes during decay. Notwithstanding, adaptations in HR occur at a considerably faster rate during HRA than with HA. There is some evidence that the molecular adaptations to heat are maintained for a considerable time via HA ‘memory’ [116], which may explain the fast re-acclimation.

### Recommendations for Future Research

This review incorporates recent studies in examining HAD and HRA. Only three of 20 studies included female participants. Given that many female athletes also compete in the heat and are eumenorrheic, studies examining HA, HAD, and HRA within this population in relation to menstrual phase are lacking. Although sex appears to be important for HA [90], the effects of sex on HAD and HRA are essentially unknown. To our knowledge, no HAD and HRA study is available that includes young and elderly participants; consequently, nothing is known on decay and re-acclimation over several age groups.

Most studies clearly specified conditions and participant characteristics, including VO_2max_ (Table 3); the exceptions were the older studies [39, 78, 98–100]. However, some studies omitted essential data, such as the first 3 days of HA [110] or the age of the participants [92]. No consensus exists regarding the use of HA regimens, making the interpretation and comparison of studies complex. For future studies, it is recommended that the basic adaptations associated with HA (e.g., *T*_c_, HR, SR) be reported for the duration of the regimen, or at least periodically with day 1 acting as the anchor for measuring adaptation. In scientific research, heat stress tests should ideally contain an element of fixed load and a component of maximal exertion so as to assess adaptations and performance parameters (e.g., Daanen et al. [93]). Moreover, HRA protocols should be sufficiently long to allow for complete return to the acclimated status. In practice, athletes may wish to conduct regular HA top-ups during the season [117].

### Practical Recommendations

Athletes who have to perform in the heat benefit from the ability of the human body to adapt to such environments. HA may be performed in natural settings and in climatic chambers under controlled conditions that simulate the hottest climate that can realistically be expected during an event (Fig. 1). Monitoring the most important physiological variables (i.e., HR, *T*_re_, and SR) until a steady state is achieved can help to determine the optimal HA duration. Considering the large inter-individual differences in HA, it can be expected that complete HA may be achieved in less than 10 days in some athletes, while others may need a longer period. Sufficient rest and cooling should be available to allow for the physiological adaptations to occur [93]. In the 2 weeks after HA, an athlete can taper to recover from the heavy training and recover from the HA regimen. During these 2 weeks a decay in HR (~ 35%), *T*_c_ (~ 6%), and SR (~ 30%) can be expected based on the derived regression equations for fully heat-acclimated participants (e.g., 10 days of daily HA, 90 min exposure, 30 °C WBGT). Exercise–heat exposure during the taper (i.e., HAD) period may help in maintaining HA status, but may also interfere with the intention of the taper to reduce exercise/training load. Heat exposure without exercise may thus be preferred during the taper [67]. When/if time allows, 4–5 days of HRA is advised during the period prior to the event in the heat, in order to achieve an acclimation status similar to that of complete HA [102]. Since the physiological adaptations in HR and *T*_c_ occur much faster during HRA, it can be expected that the decay in HR and *T*_c_ can be fully compensated for in those 4–5 days. However, the loss of adaptations for SR may be more difficult to regain in the HRA period. The period of HRA may be extended to possibly achieve an acclimation status greater than that occurring during HA [101]. This may be achieved by arriving at the competition venue ahead of time and taking advantage of the local climate to heat acclimatize. A similar HA protocol followed by HRA has recently been suggested in a study addressing the combination of training and HA [67]. Repeated HA periods were included in a preparation program for sailors and proved to be effective for maintaining acclimation status for a prolonged period of time [117]. In line with these observations, a recently published abstract of an interesting study reported that exercise–heat exposures every fifth day during the HAD period led to better sustained HR and *T*_c_ 25 days after initial HA [118]. In general, the processes should be tested prior to important competitions as they may interfere with pre-competition requirements.

## Conclusion

Quantitative analysis of 12 articles that investigated the decay in end-exercise HR, end-exercise *T*_c_, and mean SR after HA showed that adaptations in HR and *T*_c_ are lost at a rate of ~ 2.5% per decay day. It seems that a HA period of at least 5 days is sufficient to generate relatively stable adaptations in HR and *T*_c_, whereas adaptations in SR benefit from a longer period of HA. Regression analyses indicated that increasing the duration of daily heat exposure improves adaptations in *T*_c_. Exposure to higher WBGT environments during HA enhances adaptations in SR, but not in *T*_c_. Interestingly, the rate of adaptation during HRA is much faster than for the initial HA period, particularly for end-exercise HR and *T*_c_, when undertaken within a month of HA.

## Footnotes

1 Available at www.unisa.edu.au.
